# Assessment, referral and management of obstructive sleep apnea by Australian general practitioners: a qualitative analysis

**DOI:** 10.1186/s12913-021-07274-7

**Published:** 2021-11-18

**Authors:** Nicole Grivell, Jenny Haycock, Anne Redman, Andrew Vakulin, Nicholas Zwar, Nigel Stocks, Oliver Frank, Richard Reed, Ching Li Chai-Coetzer, Ronald R. Grunstein, R. Doug McEvoy, Elizabeth Hoon

**Affiliations:** 1 National Centre for Sleep Health Services Research, Bedford Park, SA Australia; 2 FHMRI Sleep/Adelaide Institute for Sleep Health, College of Medicine and Public Health, Flinders University, Box 6, Mark Oliphant Building, 5 Laffer Drive, Bedford Park, SA Australia; 3 Sax Institute, Glebe, NSW Australia; 4 Faculty of Health Sciences & Medicine, Bond University, Robina, QLD Australia; 5 Discipline of General Practice, University of Adelaide, Adelaide, SA Australia; 6 Discipline of General Practice, College of Medicine and Public Health, Flinders University, Bedford Park, SA Australia; 7 Southern Adelaide Local Health Network, SA Health, Adelaide, SA Australia; 8 Faculty of Medicine and Health, University of Sydney, Camperdown, NSW Australia; 9 Woolcock Institute of Medical Research, Glebe, NSW Australia; 10 Royal Prince Alfred Hospital, Sydney Local Health District, Camperdown, NSW Australia

**Keywords:** Sleep Apnea, Obstructive, General Practice, General Practitioners, Primary Health Care, Delivery of Health Care

## Abstract

**Background:**

The high and increasing demand for obstructive sleep apnea (OSA) care has exceeded the capacity of specialist sleep services prompting consideration of whether general practitioners could have an enhanced role in service delivery. However, little is known about the current involvement, experiences and attitudes of Australian general practitioners towards OSA. The purpose of this study was to provide an in-depth analysis of Australian general practitioners’ experiences and opinions regarding their care of patients with OSA to inform the design and implementation of new general practice models of care.

**Methods:**

Purposive sampling was used to recruit participants with maximum variation in age, experience and location. Semi-structured interviews were conducted and were analysed using Thematic Analysis.

**Results:**

Three major themes were identified: (1) General practitioners are important in recognising symptoms of OSA and facilitating a diagnosis by others; (2) Inequities in access to the assessment and management of OSA; and (3) General practitioners currently have a limited role in the management of OSA.

**Conclusions:**

When consulting with patients with symptoms of OSA, general practitioners see their primary responsibility as providing a referral for diagnosis by others. General practitioners working with patients in areas of greater need, such as rural/remote areas and those of socio-economic disadvantage, demonstrated interest in being more involved in OSA management. Inequities in access to assessment and management are potential drivers for change in future models of care for OSA in general practice.

## Background

Obstructive sleep apnea (OSA), a condition characterised by repeated obstruction of the upper airway during sleep, is associated with hypertension, reduced quality of life, increased incidence of motor vehicle accidents and stroke [1–4]. It is a highly prevalent condition, with an estimated 50% of men and 23% of women aged 40 years or over living with moderate-to-severe sleep-disordered breathing [5]. As age and obesity are major risk factors for OSA, the prevalence of OSA is likely to increase [5–7].

To date OSA has been managed primarily within a sleep specialist setting however, with increasing prevalence of OSA, the waiting times both for assessment and for care within these specialised services have increased significantly [8–10]. Since the early exploratory work of Kramer et al. [11] there has been growing interest in the role of primary care in the management of OSA [12–15]. Several randomised controlled trials have recently demonstrated that primary care management of OSA is not inferior to specialist-led care, which leads the way for a more primary care focused approach to OSA management [16–19].

General practitioners (GPs) are actively involved in the assessment and management of people with many other chronic diseases, such as diabetes or asthma. Given projected increases in the prevalence of OSA, it is possible that GPs could play a greater role in the timely assessment and management of OSA, as they do for other chronic diseases. This need is reinforced by recent work by Thornton et al. [20] that demonstrated that shorter wait times for assessment and care lead to better adherence to Continuous Positive Airway Pressure (CPAP) therapy and reduced daytime sleepiness.

Australian GPs work almost exclusively within privately-owned practices within the context of a predominantly publicly funded health care system. Activity within general practice is primarily funded by the Medicare Benefits Schedule (MBS), the funding scheme provided by the Australian Government to subsidise medical services, and within the MBS there are funding streams for comprehensive assessment and management of chronic diseases (of at least six months duration). Recently there have been changes to the MBS funding for OSA in Australia. In response to a rise in referrals for polysomnography (PSG), GPs are now required to assess individuals that they suspect of having OSA prior to patient referral for an MBS subsidised PSG: specifically, GPs must determine a patient’s likelihood of having OSA (using one of the assessment tools: STOP-BANG, OSA-50, Berlin Questionnaire) [21–23], which is symptomatic (using the Epworth Sleepiness Scale) [24, 25]. To access a PSG, Australians can refer themselves to commercial sleep services or they can be referred to private sleep physicians or public outpatient services (often with long wait times). Some commercial sleep services offer PSG with no out-of-pocket expense with the option to purchase a CPAP device following diagnosis [26]. Whilst CPAP can be funded by patients or private insurers, publicly funded CPAP therapy in Australia is restricted to those on government welfare benefits, and access is inconsistent across the states and territories of Australia [26].

It is clear from recent developments that the landscape for primary care management of OSA is shifting. The current role of Australian GPs in OSA assessment and management is unclear, as is their level of interest and/or capacity to increase their involvement in caring for patients with OSA. The aim of this study was to explore current attitudes, experiences and roles of GPs within OSA assessment and management. This study was implemented as one part of a larger project that is exploring the potential for the transition of less complex sleep services from specialist settings into primary care and was conducted to inform the development of new models of care for OSA into the current Australian general practice context.

## Methods

### Study design

This qualitative study was conducted with an inductive approach within a larger mixed-methods project. This study was designed to explore the role of GPs in managing two sleep disorders, OSA and insomnia, however this paper reports only the findings relating to OSA. The findings derived from this work related to insomnia have been recently accepted for publication [27]. This study employed in-depth interviews and thematic analysis to collect and interpret the data [28]. Thematic analysis was chosen as it encourages rich exploration of findings and supports an interpretive analysis beyond merely description [29]. Written informed consent was gained prior to participation and confidentiality was maintained throughout the study. Ethics approval was granted by the University of Adelaide Human Research Ethics Committee (ethics number H-2018-257).

### Setting

This study was conducted by interviewing practising GPs with specialist GP registration. Sampling for range emphasised variance in level of experience, socio-economic characteristics of patient population, and distance from metropolitan sleep services [30]. Participants from all six states of Australia and metropolitan, rural and remote regions were included as it was expected that participants’ interest and experience in sleep health care would vary with location and distance from specialist sleep services.

### Recruitment strategy

This study was promoted via the closed Facebook group GPs Down Under (https://www.facebook.com/groups/gpsdownunder/about), maintained and used by 8600 Australian GPs, by collaboration with local professional organisations, and by email from several members of the larger research team to professional colleagues. All research materials were provided in digital format via a short online survey prior to participants registering their interest and consenting to participate in the study. Participants were offered $150AUD reimbursement for their time commitment which is commensurate with the hourly rate of a GP. Recruitment continued until data saturation was achieved [31].

### Data collection

Twenty-eight one-on-one interviews were conducted by two researchers, EH and NG, between February and August 2019. EH is a researcher with extensive experience in qualitative methodology, data collection and analysis. NG is a registered nurse and research assistant with extensive primary care nursing experience. To promote consistency in interviewing, EH and NG regularly discussed their approach and agreed on appropriate changes throughout the data collection process. Reflecting time limitations and diverse geographical locations, all interviews were conducted by telephone, bar one which was conducted face-to-face at the participant’s request. A semi-structured interview guide was developed, guided by a conceptual framework of the barriers and facilitators influencing the implementation of complex interventions into the primary care setting by Lau et al. [32]. The guide was developed by a multidisciplinary team comprised of sleep physicians, sleep researchers, academic/practising GPs and qualitative experts. The guide was pilot tested with practising GPs and as there were limited changes made to the interview guide post-pilot interviews, these data were included in the overall sample. Interviews were audio recorded with prior consent of participants. Sequential interviewing of participants continued until data saturation was achieved which for this study was defined as when new interviews offered no new insights that could contribute towards meeting the aims of the study [30]. Participant review of transcripts was not explicitly offered, but participants were given the opportunity to provide further data or feedback via email. Interview data was transcribed verbatim by a professional transcriptionist and reviewed by the research team for accuracy.

### Data analysis

Data analysis was guided by the principles of thematic analysis, by three researchers, EH, NG and JH [28]. Interview transcripts were read and re-read to enable immersion in the data. Coding of interview transcripts was conducted using NVivo 12 software (QSR International Pty Ltd, Doncaster, Victoria, Australia), with regular meetings of the research team to discuss and refine the coding framework. Codes were derived from the data using an inductive approach. EH, NG and JH then worked collaboratively to analyse the data to identify and examine common themes and patterns within the data. Confirmation of themes and codes was conducted by re-visiting the data, both to ensure accuracy of the identified themes and to enhance rigour. Discussions about emerging themes occurred until consensus was reached, and the three themes derived from this analysis are presented in this paper.

## Results

### Participant characteristics

Of the 28 participants, 61 % (*n*=17) reported working within a metropolitan area, 32 % (*n*=9) within a rural area and 7 % (*n*=2) in a remote area of Australia. Sixty-one percent of participants were female (*n*=17) and 64 % of participants (n=18) were aged between 25 and 44 years old (see Table 1 for more detailed demographic information).

**Table 1:** Detailed participant characteristics

Participant number	Rurality	Age group	Gender	Years practising as a GP
P1	Rural	25-34	Female	3.0
P2	Rural	35-44	Male	4.0
P3	Metropolitan	25-34	Male	5.0
P4	Metropolitan	25-34	Female	2.0
P5	Rural	25-34	Female	3.0
P6	Metropolitan	35-44	Male	1.0
P7	Metropolitan	55-64	Female	29.0
P8	Metropolitan	35-44	Female	6.0
P9	Metropolitan	55-64	Female	26.0
P10	Metropolitan	35-44	Female	6.0
P11	Metropolitan	55-64	Male	35.0
P12	Metropolitan	55-64	Male	42.0
P13	Metropolitan	35-44	Male	10.0
P14	Rural	25-34	Female	5.0
P15	Metropolitan	45-54	Female	20.0
P16	Metropolitan	35-44	Female	17.0
P17	Metropolitan	35-44	Female	0.3
P18	Metropolitan	35-44	Female	3.0
P19	Metropolitan	35-44	Female	14.0
P20	Rural	55-64	Male	30.0
P21	Remote	45-54	Male	20.0
P22	Rural	55-64	Male	30.0
P23	Rural	55-64	Female	30.0
P24	Rural	25-34	Male	4.0
P25	Remote	25-34	Male	5.0
P26	Metropolitan	25-34	Female	5.0
P27	Metropolitan	45-54	Female	26.0
P28	Rural	25-34	Female	2.0

### Theme 1: GPs are important in recognising symptoms of OSA and facilitating a diagnosis by others

This first theme relates to patient assessment and referral practices, and highlights the role of GPs in supporting patients to gain an OSA diagnosis from sleep services beyond primary care. GPs generally initiated an assessment of patient’s symptoms and risk factors with a view to requesting a sleep study. Whilst some GPs noted that patients may raise the issue of sleep and tiredness, there was widespread agreement that initial assessment for OSA was primarily initiated by GPs.

*They [patient] might come in complaining of snoring or their partner complaining of snoring and noisy breathing… The second is coming in complaining of lethargy. So a vague lethargy sort of presentation. Occasionally, people come in complaining of poor sleep. I don’t generally find that. I think they generally are fairly accepting that, “That’s just the way I am”. (Participant 20)*

*Interviewer: So would the assessment of sleep apnea be initiated by you generally or the patient coming to you with concerns?*

*Interviewee: No, me, definitely. Yes. Definitely me, yes. (Participant 18)*

GPs expressed confidence in their approaches to the initial OSA assessment; they commonly identified cardiovascular and metabolic syndrome risk factors, with most, but not all, incorporating OSA screening tools into their assessment practices.

*My index suspicion, as I said, is much higher if they’re obese and especially if they sort of have that central obesity or, you know, lots of weight around their neck and sort of jowly. (Participant 8)*

Common triggers for assessment included a patient’s appearance, including weight, central obesity, and bullish necks, with one GP noting that these triggers were biased towards men rather than women. Of note, several GPs reported being less confident about assessing physical signs of OSA.

*Although I’m not super confident about diagnosing a different shaped uvula or anything, but the micrognathia, the recessive chin, is something I know to look for. (Participant 7)*

There was widespread acknowledgment of the change in MBS referral criteria for sleep studies with several GPs commenting on the impacts of this change. While one GP noted that the new referral requirements had improved their clinical practice, another noted that there may be instances where patients have risk factors for OSA without fulfilling screening tool criteria.

*So it has changed recently, … ability to refer for a sleep study. … it has actually probably been good for my clinical practice, because I think in the past I’ve been a bit ad hoc about it… “Do you snore? Are you tired?” just those really broad baseline sort of, you know, non-specific questions, but I still do that, but I…. do the proper STOP-BANG questionnaire with most patients now. (Participant 16)*

*I think the new scoring system, I think it’s fair, because … you get the odd person who is a bit neurotic and obsessed that they have sleep apnea …[but] I think there’s still people who have sleep apnea who aren’t captured by that scoring system who then aren’t eligible for the free test. I think there’s definitely people in that group … So I think there’s some people who miss – it definitely picks up the moderate to severe, but there’s some people who might have … mild to moderate sleep apnea, but including moderate where the scoring doesn’t actually pick it up, and so then they’re not eligible for a test, and they might actually benefit from treatment, but they can’t get a … Medicare sleep study. So I think there’s definitely people in that group. (Participant 27)*

Overall, most GPs described how the initial assessment led to a referral to a sleep study. GPs in this study generally understood their role as one that provides preliminary assessment for at-risk patients, and refers to sleep study services, with or without specialist input, for a formal diagnosis. As such, the majority of GPs understood their responsibility as enabling diagnosis from other service providers, who then took primary responsibility for OSA and CPAP management.

*I think our job is more as screening and getting them into the direction of the specialists and that field, whether it be from a technical point of view, practical point of view or respiratory if they’re needing to see a respiratory physician. (Participant 15)*

Several GPs reported that, prior to referral for a sleep study, they asked patients about their capacity and willingness to manage OSA using CPAP.

*I talk to them about their willingness to follow through if something was found. So if they’re up front saying there’s no way they would consider a CPAP, and there’s no way they could afford to buy a mask… or there’s another foreseeable game-changer then that would change what we do. (Participant 22)*

Many of the GPs acknowledged that, while a referral for sleep studies was straightforward for many patients, access to sleep study services or CPAP was problematic for certain groups.

### Theme 2: Inequities in access to the assessment and management of OSA

This theme explores inequities of access to both diagnosis and treatment of OSA within the Australian health system. GPs reported challenges in accessing sleep studies and CPAP therapy related to both socioeconomic and geographic factors. Many GPs reported long wait times for public sleep services.

*I know there is public system…but I know they’re not going to get seen in the next…12 to 18 months…there’s a huge wait list in the public system… who knows when and where they would get an appointment. (Participant 15)*

*If I didn’t have [the option to refer privately], it would be terrible, particularly the public clinic. It just takes forever, absolutely forever to get people seen. (Participant 16)*

GPs located in rural and remote areas also reported challenges in finding suitable referral options for sleep health care, stating that there were limited options available outside metropolitan areas. These GPs also reported that their patients had difficulties accessing metropolitan based sleep services because of distance, with some patients being located up to 800 km away.

*So we don’t have any local respiratory physicians, so my options are to refer people, if I wanted to do a formal sleep study, [to] a respiratory physician. They have to be referred sort of 200 km away, there’s two spots that I could send them to, but that requires the financial means to get there…The nearest [public] sleep clinic to us is 500 km away. Not even practical [to refer patients]. (Participant 23)*

*The only one we can refer them to is a respiratory specialist in Townsville, which is 800 km away… They have to go 800 km to get a sleep study. (Participant 25)*

The inequities within OSA management were not restricted to assessment: GPs also expressed concerns about access to treatment. Participants reported concerns that some patients living on low incomes could not afford CPAP machines yet were ineligible for publicly funded CPAP, leaving their patients without treatment for their OSA.

*The issue would be cost. So some people would like to, but they can’t afford the machines, so the machines cost around 2000 – two to three thousand [dollars], depending on what you get, so some of the patients would like to, but they can’t afford it, so they still have sleep apnea and it’s untreated. (Participant 21)*

*The problem is if they actually get to the stage where they do need the sleep apnea machine, only the very – the most severe of sleep apneas will qualify for Enable, which is a New South Wales health scheme to co-fund…but…even then if they’re eligible, it takes about six months on the waiting list to get a machine…and most people don’t qualify for it… the machines are $5000 and they are people on Newstart or disability pensions, $240 a week, paying living and food, they just – there’s no hope of even saving up for one. (Participant 27)*

*When I know I can prevent someone getting heart disease and strokes by giving them CPAP, but they can’t get CPAP, because they can’t afford it, it’s pretty frustrating. (Participant 28)*

Of note, one participant reported that the financial means of a patient was a factor they considered when deciding whether or not to progress towards an OSA diagnosis. Others expressed frustration at not being able to provide optimal care for those patients that are unable to afford CPAP therapy.

*The decision to refer [for a sleep study] would be based on where the patient is, the patient’s financial status and…what my intent was. So at that point, I would ask them whether they were interested and prepared to look at management of the obstructive sleep apnea if we found it. (Participant 23)*

*If they can afford the CPAP, they are usually really adherent, because they can actually see a really big benefit really quickly, but it’s the patients who just can’t afford it. That’s when you tend to run into trouble. (Participant 16)*

Many GPs reported that they refer to commercial sleep providers as an alternative to the lengthy wait times for public services however they understood that this is dependent on patients having the financial means to purchase CPAP if it is recommended by the private provider. Coupled with pressure to refer to these commercial services was some uncertainty about the ethics of these private clinics and potential conflict of interest associated with offering both a diagnosis of OSA and an option to purchase a costly CPAP machine. This prompted GPs to initiate ‘work arounds’ to relieve the pressure on their patients to purchase CPAP machines after the sleep study, such as supporting patients to purchase CPAP devices online.

*So it’s difficult, but I think private is more efficient and, yes, it costs money, but if patients can afford it and wants to give it a go I – yes, I would encourage them to use private providers. (Participant 17)*

*There are some concerns that patients have expressed in the past that the – most clinics tend to be very pushy towards buying sleep apnea machines off them… offering packages, servicing…which I’m a little bit uncomfortable about, so I usually tell them that once they have a diagnosis and once they have their chat with the sleep clinic tech…I tend to ask them to come back, because patients of mine have found it cheaper to purchase CPAP online. (Participant 13)*

### Theme 3: GPs currently have a limited role in the management of OSA

This theme explores the concept of accountability and responsibility for the ongoing management of patients with OSA. When discussing the care that they provide to patients who have been diagnosed with OSA and have commenced treatment, GPs commonly described a role that was limited to the management of associated co-morbidities. However, this was not the case for all GPs, with interest in becoming more involved and having greater accountability for OSA management more commonly identified by participants who identified a distinct need within their patient cohort, such as in non-urban settings or areas of social disadvantage.

As described in Theme One, many GPs considered their role in OSA as assessing patients that they suspect have OSA and organising a referral for a sleep study for diagnosis. Following receipt of a diagnosis, most GPs then considered their responsibility as providing care for associated co-morbid conditions such as obesity and cardiovascular risk factors, and organising a referral to a specialist physician.

*So, generally speaking, I haven’t been doing much of the management myself…if they’ve got moderate/severe sleep apnea I will often get them to go see a sleep specialist. If they’re kind of like mild obstructive sleep apnea I will try a lifestyle thing, like reducing alcohol intake, losing weight, things like that. (Participant 24)*

Some participants did recognise treatment options for OSA other than CPAP, such as weight loss for mild OSA or mandibular advancement splints, but most commonly GPs associated the treatment of OSA with CPAP therapy.

*I think we tend to send less people to ear, nose and throat for the splints and the – that was more of a treatment say five to 10 years ago, and we don’t seem to do that anymore, but I think that’s probably because there has been all these advances in CPAP and stuff like that that they tend to need it. (Participant 16)*

*My understanding is the most effective strategy, and certainly my experience has been, CPAP. (Participant 8)*

Most participants were not actively involved in the ongoing management of CPAP, with some stating that patients instead saw a specialist physician periodically for review or were reviewed by the practice nurse. Of note, one GP was uncertain as to who was providing the ongoing CPAP management for their patients.

*So I don’t provide any ongoing management… Most of the people that I have who are using CPAP machines would be looking at intermittent follow up with respiratory physicians from where they got their initial prescription. (Participant 23)*

*Often it’s not even seeing them [myself]. Like in seeing the nurse who will check that the CPAP machine is working okay. (Participant 8)*

*If they think their mask is leaking or their tubing is not quite right or they can’t the nose piece to fit on properly, they will go and see – there’s someone through – but, yes, I don’t quite know who that someone is. It depends on the company that they’ve ended up buying the CPAP machine through…if they’ve got a problem they go back to them, and they will do a printout and see what their pressures have been like. But that bit, I don’t really have that much to do with. (Participant 15)*

Some GPs reported that they believed that review of CPAP therapy wasn’t within their scope of practice, instead that it was the responsibility of the clinic initiating CPAP therapy or the specialist physician.

*I don’t get very involved in [CPAP] – and I’ve never sort of looking at the data download from those – the pumps that capture pressure data and things…if the person comes back to me with concerns that it no longer seems to be working or they’re having trouble with their mask I would generally send them back to the people who are dealing with masks and fittings and pump pressures all the time, and that’s generally the technicians…I’m not going to take on, you know, how to fit people’s masks and how – and that sort of stuff…I’m not going to take on that technician role. (Participant 22)*

*[CPAP management is] sort of similar to what people have now with pacemakers when their pacemaker gets monitored, and it goes back to cardiologist. I would never see that in general practice…It would only ever go to them, because then if something had to be done they could adjust those settings, whereas if I got that information I wouldn’t know how to adjust their settings…So I would prefer not to get that… Otherwise it’s out of my depth. (Participant 26)*

In contrast, one participant who worked in a rural practice felt that the management of OSA should largely be the responsibility of primary care. They expressed a belief that GPs could have a greater role in the assessment and ongoing management of OSA.

*I think it is an area that – I think a lot of sleep apnea is actually a primary care issue, not a specialist issue… and I think we need to consider ways that – because it is so common,… we can involve more primary care physicians and health care workers in that sort of identification management and monitoring phases. (Participant 20)*

Several participants described reasons other than scope of practice for not being more involved in CPAP management. Reasons included lack of knowledge about CPAP, lack of funding to support time to analyse CPAP data downloads and lack of infrastructure to provide comprehensive OSA management within primary care. For others, limited involvement in CPAP management reflected a lack of interest.

*I generally would ask how they’re going, how they’re coping with the CPAP, are [they] tolerating it, but I have not in terms like adjusting the CPAP or anything else. I just don’t really know enough about it. (Participant 4)*

*If we’re just talking about looking at output from the machines, and so on, there’s an issue for GPs partly [with] sheer volume of data, like how much stuff are we going to look at, and Medicare does not pay for us to look at it unless the patient is sitting there with us. (Participant 12)*

*Generally, it goes back to the physician, because there’s not really any way to monitor it in primary care. (Participant 20)*

*I have zero interest in checking someone’s CPAP machine. Like I feel like that’s a task that’s done best by a technician. (Participant 8)*

Conversely, one participant had taken a special interest in OSA management, prompted by lack of sleep services accessibility. The GP described a new model of care that he had instigated to support patients to access much needed diagnostic services and ongoing management in his remote location.

*I just refer them to my practice nurse, who does the sleep study, and then we send the digital recording to a sleep specialist in Adelaide, and they report on it… And they bulk bill for looking at it, and we charge them for using our machine, so they get billed. When I send them for a sleep study we bill them, because it costs time and money to have the machine here and also the nursing staff to set it all up and then the actual interpretation of the recording is bulk billed by the private sleep specialist in Adelaide… We see [the patient] a week later with the results. I get a written report from the specialist, which will give a number of recommendations… and then we follow that pathway. (Participant 21)*

The level of interest in becoming more involved in OSA management varied between participants. Some GPs expressed enthusiasm for becoming more involved, and this was more commonly expressed by those participants working in rural and remote locations, prompted by a lack of accessible local sleep services, and those working in low socio-economic areas.

*I think, as a rural GP, we are pretty keen to be as involved, you know, do as much as we can for our patients when services might be distant. (Participant 24)*

*I think I feel constricted around funding and getting things organised in a low socio-economic area, but I would be happy to play more [of a] role in managing, treating sleep apnea going forward if there was the right training. (Participant 13)*

A number of factors were identified by GPs as impacting on their ability to be more involved in the management of their patients with a diagnosis of OSA. Participants reported that a lack of knowledge and training, and inadequate funding were barriers to greater involvement in OSA management.

*I wouldn’t mind being upskilled in how to manage it, and then I could follow them up myself…That would be great. You know, if I had some idea about what the numbers meant and, you know, could look at people’s downloads and adjust for them…I would super happy to be involved in that, but it’s just not something we know about, so I can’t provide it safely at the moment. (Participant 28)*

*If there is funding and expert’s training, I’d be happy to play a bigger role going forward for the future. (Participant 13)*

*If I can get an item number as a GP… I could actually read the sleep study and say, “Yes, you’ve got sleep apnea. Let’s put a mask on and see how it improves,” and do that without having to send someone to the nearest sleep physician or respiratory physician would be fantastic. (Participant 25)*

*I think GPs, you know, are ready and willing to take responsibility and more of it in some cases, as long as somebody adequately is, again, paying for their time to do it. You know, a big…problem is the system does not offer adequate payment or no payment at all in many cases for things that we’re supposed to do. (Participant 12)*

## Discussion

This qualitative study aimed to provide an in-depth understanding of Australian GPs’ current assessment and management practices for OSA and to identify factors that are likely to either facilitate or impede change to the clinical care of OSA. This detailed analysis is designed to inform the current drive within the Australian health system to implement clinical and cost-effective interventions to enhance the role of primary care in OSA assessment and management. The three dominant themes identified in this study raise important issues about the factors that influence current clinical practice and the potential to implement new models of care. Lau and colleagues’ conceptual framework [32], which describes the key elements (related to external context, organisation, profession and intervention) that influence implementation of change within general practice, provides a useful frame to further interpret the study’s dominant themes and are discussed below.

### External context influencing GP practice

It is clear that a number of Lau et al’s [32] external level factors (identified in italic) influence current GP practice related to OSA. Limited, publicly-funded OSA diagnosis and treatment options including CPAP, and consequential lengthy waiting lists (*governmental financing*) has been countered by a concurrent growth in commercial sleep services which provide pathways for OSA diagnosis and CPAP treatment for patients who can afford the ‘out of pocket’ expenses tied to these commercial services (*economic climate*). For GPs who care for patients on low incomes or those living in rural and remote communities (*infrastructure*) the lack of accessible OSA diagnosis and affordable treatment options may be a driver of change, and a point of intervention to enhance the role of primary care in OSA diagnosis and management.

The thematic analysis also highlighted how the *dominant paradigm* that OSA, whilst investigated by GPs, is diagnosed and managed by others, influences not only the GPs’ focus on initial assessment processes but also the swift referral to other professionals to complete sleep studies, and where appropriate, initiate and monitor CPAP treatment. The recent regulatory change to MBS funding in Australia for sleep studies reinforces the OSA assessment role for GPs, through the requirement of GPs to use validated OSA assessment tools to initiate publicly funded sleep studies. For some GPs, relationships with specific commercial CPAP providers (*inter-professional relationships*) reinforce commercially based care pathways. Even where GPs acknowledge a potential conflict of interest in recommending specific commercial CPAP providers, the paucity of alternative public services is a barrier to GPs offering other referral options to patients. In line with other studies [33, 34] some GPs find ‘work-arounds’ to support their patients’ access to cheaper CPAP options, such as online purchases, and demonstrates that some GPs are motivated to overcome external contextual barriers to deliver affordable CPAP treatment.

### Organisational factors

One of the key organisational factors that influence GPs’ clinical practice for OSA is the way current pathways encourage referrals to other dedicated professionals (sleep specialists and sleep technicians) who take responsibility for diagnosis, CPAP treatment and monitoring (*processes and systems*). This dominant way of managing the diagnosis and CPAP monitoring components of OSA fits efficiently with GP’s busy workflow and extensive clinical responsibilities (*available resources*), so that their limited time in consultations may be focussed on clinical management of other OSA comorbidities. Currently, almost all GPs do not have access to *technical support* from staff within their practices for OSA diagnosis and CPAP treatment, while many do have access to specialist and technical support from outside professionals. Of note, some GPs identified that practice nurses could provide some of the technical support for CPAP treatment within the primary care setting in the future.

### Professional factors

As found in the review by Lau et al. [32], there are a number of professional factors influencing current and potential future clinical practice of GPs. The thematic analysis identified that *lack of confidence* in the interpretation of CPAP reports and limited *professional interest* in the technical aspects of CPAP treatment delivery, influenced how GPs engaged with OSA management beyond diagnosis. It is also of interest that there was little discussion about treatment options for OSA beyond CPAP. There was also lack of *awareness* about the real cost of CPAP expressed by several GPs, with an inflated perceived cost potentially leading to GPs’ discouraging CPAP therapy for those patients with severe OSA for which CPAP is the preferred treatment. Overall, there was limited interest to expand their scope of practice (*professional role*) to CPAP treatment, unless the GP was providing care for patients living in rural and remote areas or who were economically disadvantaged, and therefore unable to access alternate OSA diagnostic and CPAP services. For these GPs, their *relationship with their patients* and their underlying view of their primary care role (*philosophy of care*), were driving factors in their interest in having greater involvement in OSA management and specifically CPAP monitoring. Additionally, there were a few GPs who expressed a professional interest to expand their knowledge of and competence in CPAP and OSA management. This fits with the trend within Australian primary care for some GPs to become GPs with Special Interest for specific conditions [35, 36], and this may be a potential point of intervention to enhance the role of primary care in OSA management.

### Interaction of these contextual factors

The dominant themes and interpretation of these themes using Lau et al’s [32] conceptual framework highlights how both current GP clinical practice and options to develop new models of care will be influenced by the interaction of multiple contextual factors (at external, organisational and professional levels), with some factors being drivers and others being barriers to change. This study identifies how these factors influence care of OSA and provides examples of how these factors interact for GPs in specific practice settings, which is an important consideration when designing and implementing interventions for OSA in the Australian setting [37].

### Strengths and limitations

A key strength of this qualitative study was its use of appropriate research strategies and techniques to ensure rigour, including purposeful sampling, consistent data collection techniques, peer review and ongoing analysis using triangulation by multiple researchers to develop preliminary findings and working to gain consensus on final themes [38]. The inductive approach to data collection and analysis emphasised perspectives of participants with thick description, and the use of the conceptual framework by Lau et al. [32] in the interpretative stage provided an opportunity to compare the study’s findings with a wide range of other studies that have explored the key factors that influence change in primary care. Whilst it is recognised that this study has examined the assessment and management of OSA within the specific contextual setting of Australian primary care, and that there are therefore limits to the transferability of these findings, it is anticipated that learnings from this study will have relevance for other countries with similar primary health care systems. However, it is recognised that, despite reaching data saturation and the use of purposive sampling to recruit participants with varied experience and rurality, with a limited number of remote participants the sample is not fully representative of all practising GPs within Australia. It is also acknowledged that this study was completed at a specific point of time, and factors influencing care may rapidly change, for instance in health pandemics such as COVID-19. These contextual factors were not captured in this analysis. One notable exception is the exploration of telemedicine in the management of OSA within general practice. The COVID-19 pandemic has brought additional challenges to the provision of care. Limits to face-to-face consultations has resulted in a greater demand for telemedicine, with the Australian Government providing temporary support for the use of telemedicine to reduce transmission of COVID-19 within the Australian community [39]. With recent research demonstrating the effectiveness of telemedicine in improving CPAP adherence in patients with OSA, it may be that Australian GPs could conduct screening for OSA, using the standardised OSA screening tools, and monitor ongoing OSA management via telemedicine [40]. This could also be of benefit to those individuals living geographically distant from their health care providers. As the role of telemedicine within general practice management of OSA was not explored within this study, it offers an opportunity for this topic to be explored in depth within future research.

## Conclusions

It is apparent from this study that for there to be fair and equitable access to the assessment and management of OSA there is a need for change amongst those living in rural or remote locations and/or in areas of socio-economic disadvantage, and that this need is well recognised by GPs. This study demonstrates that, whilst there are external, organisational and professional level barriers to change, there are also drivers supporting the implementation of new models of care for the primary care management of OSA. It is important to note however, that any enhanced assessment and management of OSA by GPs would be limited without widespread access to CPAP. By providing GPs with the skills, knowledge and infrastructure to deliver more comprehensive assessment and management of OSA, GPs may be empowered to address the needs of patients living with or suspected of having OSA, irrespective of their geographic location or socio-economic status.

## Data Availability

The datasets generated and/or analysed during the current study are not publicly available due to concerns participant privacy may be compromised but are available from the corresponding author on reasonable request.

## Abbreviations

OSA: Obstructive sleep apnea
GP: General practitioner
CPAP: Continuous positive airway pressure
MBS: Medicare Benefits Schedule
PSG: Polysomnography
